# Comparison of salivary caspase-3 biomarker in peri-implantitis among type 2 diabetic patients

**DOI:** 10.6026/973206300222405

**Published:** 2026-04-30

**Authors:** Madhu Kiran, Awadesh Kumar Gupta, Deepak Bala, Gopinath Parakkat Vynat, K. Rajesh Kumar, Manjunath S.M, Syed Fareed Mohsin, Bassam Mohammed Almutairi

**Affiliations:** 1 Department of Conservative Dentistry and Endodontics, Sri Venkateswara Dental College, Bannerghatta, Bengaluru, Karnataka, India; 2 Department of Oral Pathology and Microbiology, DJ College of Dental Sciences and Research, Modinagar, Ghaziabad, Uttar Pradesh, India; 3 Department of Periodontology, Rayat Bahra Dental College and Hospital, Mohali, Punjab, India; 4 Department of Periodontology and Implantology, Sree Anjaneya Institute of Dental Sciences, Kozhikode, Kerala, India; 5 Department of Prosthodontics, Malabar Dental College, Kerala, India; 6 Department of Oral and Maxillofacial Pathology, Dental College & Hospital, College of Dental Sciences, Jammu, India; 7 Department of Oral and Maxillofacial Diagnostic Sciences, College of Dentistry, Qassim University, Buraydah, Saudi Arabia; 8 Department of Oral and Maxillofacial Surgery, College of Dentistry, Qassim Univesrity, Buraydah 52571, Saudi Arabia

**Keywords:** Caspase-3, diabetes mellitus, enzyme-linked immunosorbent assay, peri-implantitis

## Abstract

Diagnostic biomarker-based methodologies have become the form of reliable non-invasive measures of diagnosis of peri-implant disorders at the earliest possible stage. Therefore, it is of interest to determine efficacy of salivary caspase-3 levels as a non-invasive biomarker of early peri-implantitis (PI) diagnosis in diabetic patients with type 2 diabetes. The number of participants was 36, which was separated into three groups. Group I- PI with the presence of the diabetes mellitus (DM + PI) and group II- PI without the diabetes mellitus (NDM + PI) and group III- periodontally healthy and no diabetes mellitus (NDM + PH). Each of the participants had unstimulated whole saliva (10 mL) collected. Centrifugation and Salivary caspase-3 levels quantification was done on samples by using a commercially available Human Caspase-3 enzyme-linked immunosorbent assay kit. The data obtained was statistically tested. Caspase-3 has the potential of being a non- invasive salivary biomarker in high-risk groups.

## Background:

Missing teeth are replaced using dental implants. The effectiveness of implants is related to the underlying medical condition and local factors. The contribution factors towards the implant success include local factors like the type of implant, free of infection and bone type [1, 2, 3, 4-5]. Peri-implantitis (PI) is a progressive inflammatory problem with regard to the soft and hard tissues surrounding dental implants. It usually causes failure of the implant when untreated or not diagnosed. This disorder is more risky in patients who have systemic conditions especially type 2 diabetes mellitus. This impairs the immune reaction and hastens the inflammatory and apoptotic mechanisms [6]. As the number of full- arch implant-supported prostheses among diabetic patient's increases, there is an urgent need to identify credible diagnostic predictors [7]. Peri-implant infection is differentiated into Peri-implant mucositis (PM) and peri-implantitis (PI) [8]. Various microorganisms are involved in the peri-implant inflammation [9]. Peri-implantitis diagnosis is done by the traditional method that encompasses bleeding on probing (BOP), periodontal pocket depth (PPD), mobility, and bone loss measurements. Cytokines, interleukins present in the gingival crevicular fluid (GCF) and Peri-implant crevicular fluid (PICF) released after bone destruction and inflammation can be used as a specific and early parameter of peri-implantitis. Some of the biomarkers that have received most attention of the medical researches include the cytokines which are inflammatory mediators and play a crucial role in the development and progression of various autoimmune, infectious and inflammatory diseases. The cells of adaptive and innate immunity secrete soluble proteins, known as cytokines [10]. The treatment of peri-implant illnesses is normally combined using non-invasive, invasive and pharmaceutical interventions [11]. Saliva has been receiving the interest as a promising diagnostic fluid because of its non-invasive collection and easy manipulation, and its high content of biomarkers that indicate the local and systemic health [12]. Salivary biomarkers including glucose, interleukins, tumor necrosis factor-alpha (TNF-alpha), and advanced glycation end product have been considered as important in the inflammatory response and tissue destruction in diabetes patients [13]. Most likely, of such proteins, caspase-3, which is a major effecter protein in the apoptotic cascade, has become a possible surrogate endpoint since it plays a central role in cell death and tissue turnover in inflammatory states [14]. Caspase-3, a proteolytic enzyme, also known as the executioner caspase. The protein responsible of cleavage of a variety of intracellular proteins during apoptosis. It has been shown to be highly expressed in periodontal tissue breakdown and PI inflammation [15]. The caspase-3 caspase-3 is found in saliva providing a readily available, noninvasive method of measuring oral inflammatory state of high- risk patients, including diabetic patients [16]. Salivary diagnostics have been promising in early diagnosis and disease follow-up, and these suits well with the normal dental practice [7]. Therefore, it is of interest to determine the salivary caspase-3 as a non-invasive biomarker to early detect and monitor PI in type 2 diabetic mellitus patients.

## Materials and Methods:

This was an observational study that was conducted in the Oral implantology department. The research was conducted following the consent of the participants and informed consent of the concern authority. The study was included with 36 participants that were in line with the inclusion and exclusion criteria (healthy and diabetic patients between the ages of 35 and 55 years, both genders, no other systemic conditions). The participants were screened and put into the categories of diabetic (HbA1c ≥ 6.5%) and non-diabetic based on their systemic condition. Additional screening of each case involved the presence or absence of PI. The researchers conducted the study between February 2022 and November 2025. Those who were divided into three groups (n = 12 each) based on glycemic status (HbA1c ≥ 6.5% in diabetics) and presence of PI (probing depth ≥ 6 mm and radiographic bone loss more than one thread) constituted 36 participants. Group I- PI patients with presence of diabetes mellitus (PI+DM), group II - PI with no diabetes mellitus (PI+NDM), and group III - periodontally healthy and free of diabetes mellitus (healthy control group). Whole saliva (10 mL) was collected at 5 minutes in sterile polypropylene tubes when it was not stimulated. The samples were centrifuged and the concentration of salivarial caspase-3 was measured through commercially available ELISA kit of Human Caspase-3 enzyme-linked immunosorbent assay kit. This was followed by the incubation of these micro plates with saliva samples taken out of the study subjects so that the caspase-3 could attach itself to the antibodies. IBM Statistical Package for the Social Sciences version 24.0 was used in the evaluation of the obtained data in a statistical manner. It was analyzed by ShapiroWilk test, KruskalWallis test, Chi-square (X2) and MannWhitney test.

## Result:

The number of participants was 18 males and 18 females. Table 1 (see PDF) indicates the level of salivary caspase-3 in the three groups. The highest mean salivary caspase-3 level was found in group I compared to Group II and lastly healthy groups. It describes the significance of salivary biomarker in the identification of risk group.

## Discussion:

Dental implants have transformed restorative dentistry by providing a long lasting and aesthetically better option to the conventional prosthetics. Peri-implantitis, which consists of gradual inflammation of soft and hard tissues around the implant, is the condition that occurs in most of the implant patients, 5 to 10 years after post-placement [17]. Existence of diverse biomarkers assists in early determination of peri-implant inflammation. Saliva can be developed into a diagnostics and monitoring system of several diseases. A prominent effector enzyme of the cascade of apoptosis, caspase-3, assumes a central role in cell death and inflammatory reactions [18]. The results of the current study have shown a statistically significant increase in the levels of salivary caspase-3 in the patients with the peri-implantitis (PI), especially among the patients with type 2 diabetes mellitus (PI) in comparison with the nondiabetic patients and controls. Higher caspase-3 levels in diabetic patients with PI in our study indicate higher levels of apoptotic activity and poor tissue homeostasis thus it is a good diagnostic tool. Fathima *et al.* evaluated salivary caspase-3 as a simple biomarker of the detection and monitoring of peri-implantitis at its early phases. They made the conclusion that, PI was positively correlated with elevated salivary caspase-3 levels, in particular, in patients with type 2 diabetes mellitus [7]. Our result goes hand in hand with the findings. Higher levels of caspase-3 observed in systemic diseases such as diabetes, in gingival crevicular fluid and serum of periodontitis patients [19, 20]. This showed that periodontal tissue status and inflammation can be well and effectively reported with salivary biomarkers such as caspase protein-related substances [21]. Saxena *et al.* were able to evaluate the degree of interleukin in peri-implantitis diagnosis. They made a conclusion that, a diagnostic tool can be used as biomarker [10]. According to Bahadur *et al.* the use of different salivary biomarkers can be used to detect changes of inflammatory in the area of implant periapical [3]. Nayak *et al.* discovered that, salivary MMP-8, IL-1 and TNF- alpha had a high potential of being reliable and non-invasive biomarkers in early detection and monitoring of peri-implantitis [22]. Manek *et al.* made a conclusion that, endothelin-1 and interleukin-1b biomarkers are useful in peri-implant disorder diagnosis [8]. According to Narayankar *et al.* NTx and calprotectin were highly found in peri-implantitis group [23] La Monaca *et al.* concluded, based on Meta-analysis, that biomarkers play a moderated role in the diagnosis of peri-implantitis [24]. Sharma *et al.* reasoned that the level of salivary titanium ion concentration rose depending on the severity of peri-implant disease and was related to the clinical signs of inflammation and bone resorption [17]. Peri-implant marginal bone loss is underestimated in periapical radiograph due to the fact that they capture the mesial and distal images of implants and not 3D morphology of bone defects [24]. The radiographic bone loss must have a bone loss of at least 40 percent to detect resorption. Therefore biomarkers are useful in early identification of initial peri-implant inflammation which is non-invasive operation. The positive features of this study are that it is based on a non-invasive salivary biomarker method that allows an easy and convenient collection of samples of high-risk, totally edentulous diabetic patients. The rigor of method is improved by the use of validated ELISA protocols and well defined clinical and systemic criteria. Study limitations include; the research design does not allow the researcher to determine the cause and effect between high levels of caspase-3 and PI. The research should be in future seeks to undertake a prospective study with the larger sample sizes and diverse population to confirm these results. Advancement to knowledge with this research is the salivary caspase-3 levels were significantly higher in diabetic patients with PI. Salivary caspase-3 levels are associated with peri-implantitis.

## Conclusion:

We show that there is a strong correlation between high salivary caspase-3 levels and PI in the type 2 diabetes mellitus patients. To increase the oral health and the overall results of implants in diabetic patients, better clinical guidelines and additional studies are needed.
